# Supplementary figures and images for: Sex-dependent development of Kras-induced anal squamous cell carcinoma in mice
Source: PLoS One. 2021 Nov 4;16(11):e0259245. doi: 10.1371/journal.pone.0259245 (PMC8568287; doi:10.1371/journal.pone.0259245)

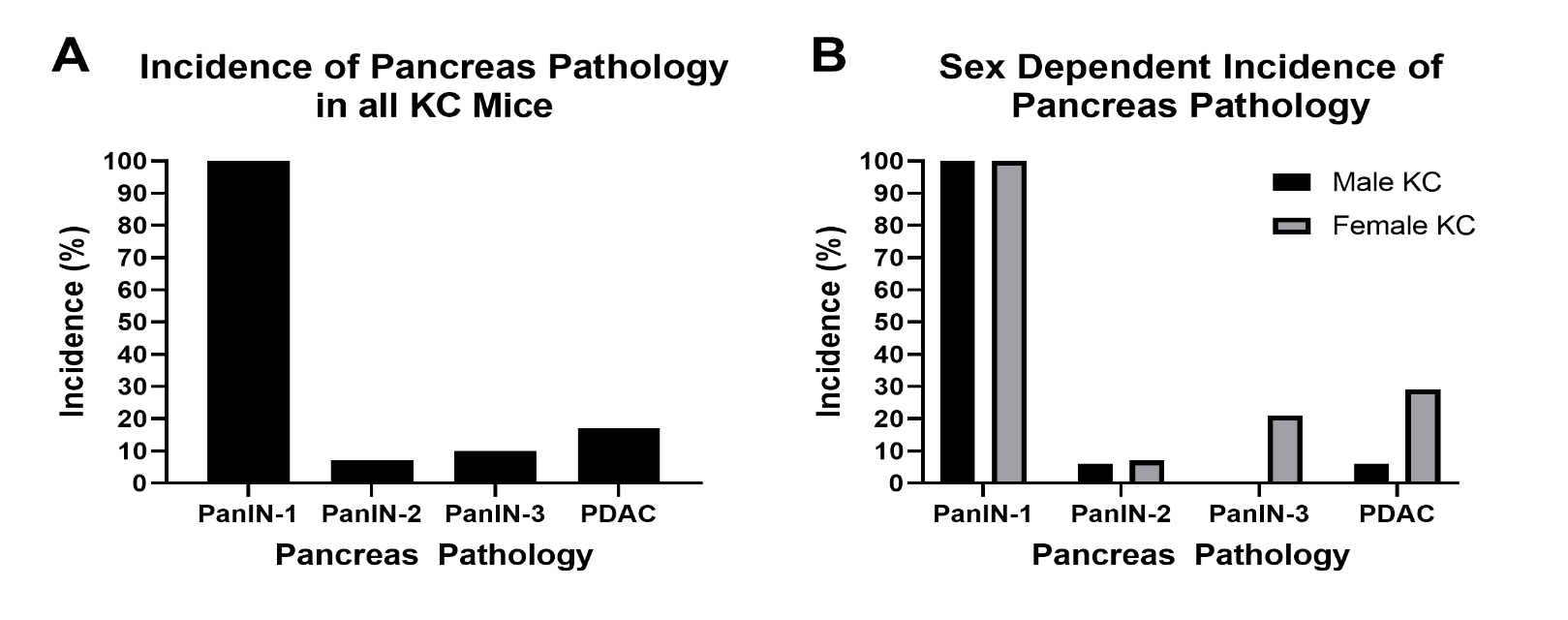

Supplement: S1 Fig — A) The incidence of pancreatic precursor lesions (PanIN-1, PanIN-2, PanIN-3) and PDAC in all the KC mice match what was previously reported in this model. B) There is no statistically significant differences in development of pancreatic pathology between male (n = 16) and female KC (n = 14) mice. Both males and females show a 100% incidence of PanIN-1 (p-value = 1). PanIN-1, PanIN-2, PanIN-3 and PDAC male vs female incidence with their p-vales are as follows: 100% vs 100% (p = 1), 6.25% vs 7.15% (P > 0.99), 0% vs 21.43% (P = 0.09) and 6.25% vs 28.57% (P = 0.16). (TIF) [file pone.0259245.s001.tif]

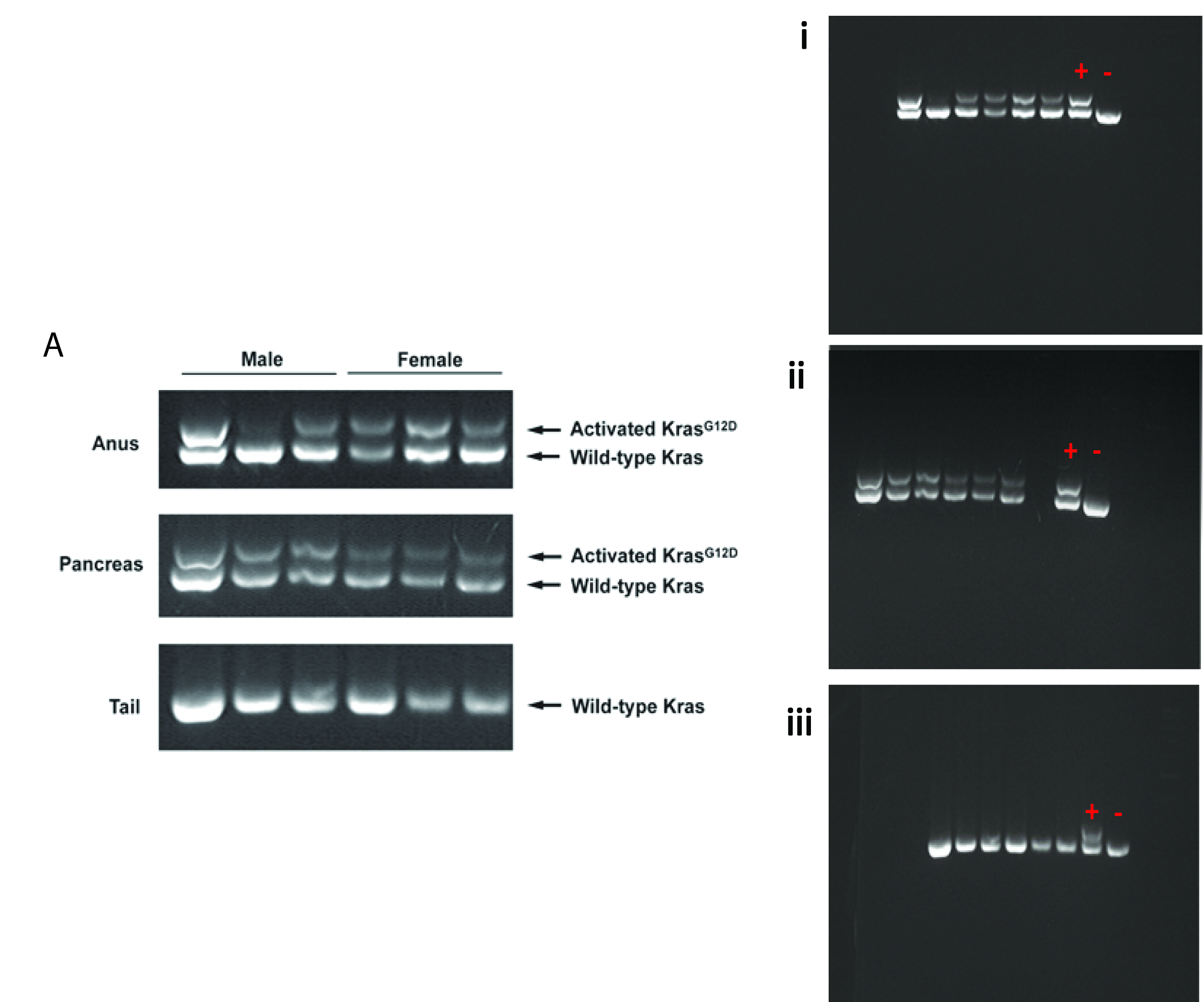

Supplement: S2 Fig — Full gel images of mutated Kras genotyping in 9 month old male and female anus (i), pancreas (ii) and tail (iii) next to the cropped image used in the manuscript text (A). The ‘+’ and ‘-‘ indicate the positive and negative control bands on each gel. The positive control is DNA from LSL Kras/+; Mx-1 cre/+ mouse bone marrow containing the activated KrasG12D gene (Control (+)) and the negative control is DNA from the pancreas of a C57BL/6J mice (Control (-)). (TIF) [file pone.0259245.s002.tif]

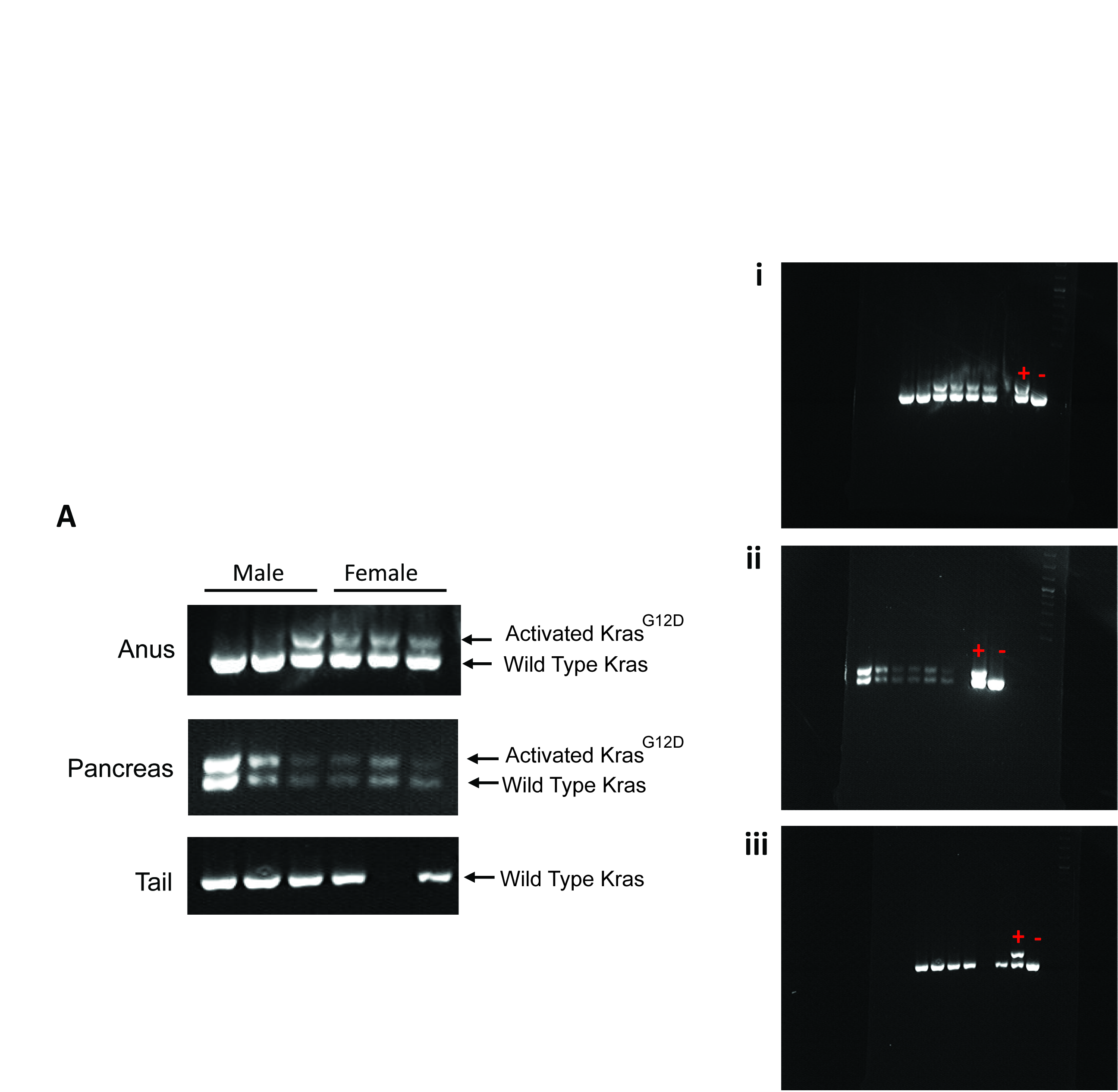

Supplement: S3 Fig — A) Full gel images taken of the PCR product. This shows the male KC anus with the activated mutant Kras gene at an early age (8 weeks old). Despite the active mutant Kras gene being present, no males develop anal SCC. The full gel images for the anus (i), pancreas (ii) and tail (iii) are to the right of the full image. The ‘+’ and ‘-‘ indicate the positive and negative control bands on each gel. The positive control is DNA from LSL Kras/+; Mx-1 cre/+ mouse bone marrow containing the activated KrasG12D gene (Control (+)) and the negative control is DNA from the pancreas of a C57BL/6J mice (Control (-)). (TIF) [file pone.0259245.s003.tif]

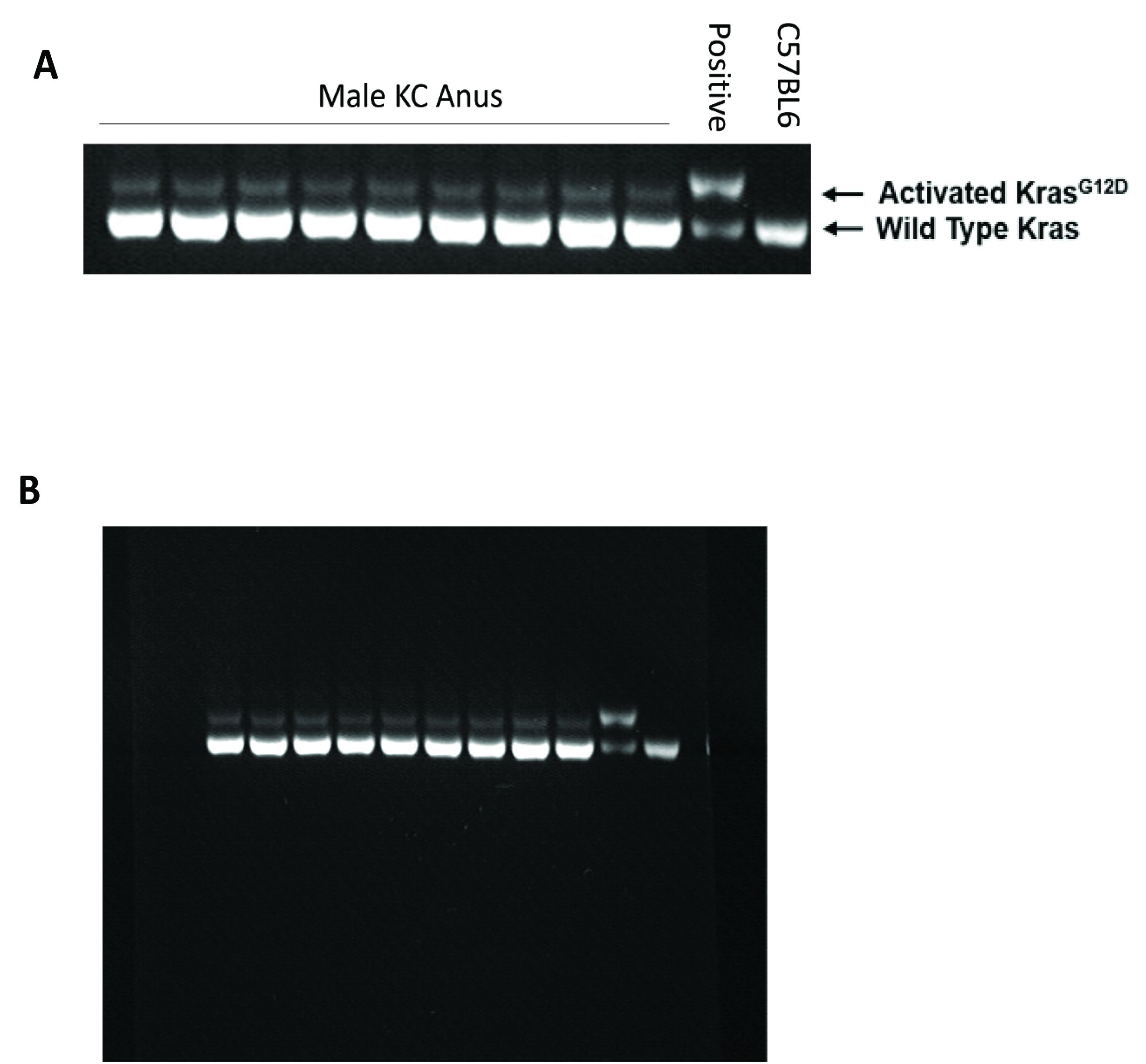

Supplement: S4 Fig — Genomic DNA was isolated from the available FFPE samples from age 9 month male KC mice and analyzed using PCR for the activated KrasG12D mutation. All male anus showed the presence of the activated KrasG12D mutation within the anal tissue (A). The full gel is shown in panel B. (TIF) [file pone.0259245.s004.tif]

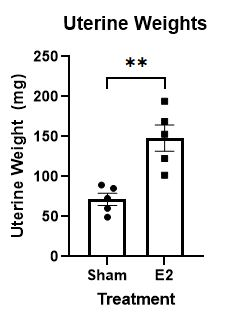

Supplement: S5 Fig — The E2 dosed female mice have a significantly increased uterine weight compared to the sham dosed mice indicating successful E2 administration. (TIF) [file pone.0259245.s005.tif]
