# Supplementary material for: Sex-dependent development of Kras-induced anal squamous cell carcinoma in mice
Source: PLoS One. 2021 Nov 4;16(11):e0259245. doi: 10.1371/journal.pone.0259245 (PMC8568287; doi:10.1371/journal.pone.0259245)

Raw Images for Figure 6 (also shown in supplemental figure 2)
-The supplemental figure is included next to the raw images for clarity as to which part came from which raw gel.

Raw Images

i

Anus

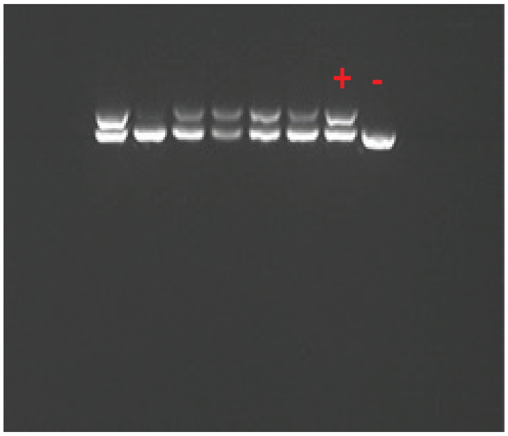

ii

Pancreas

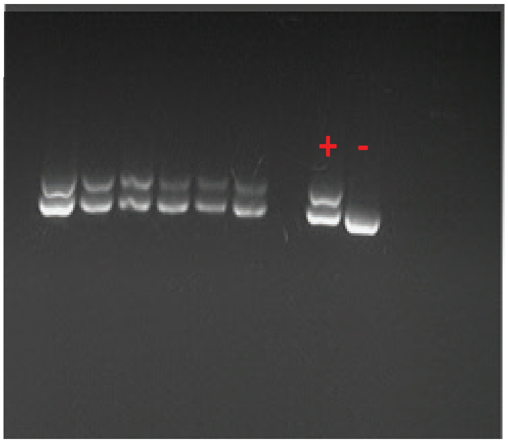

iii

Tail

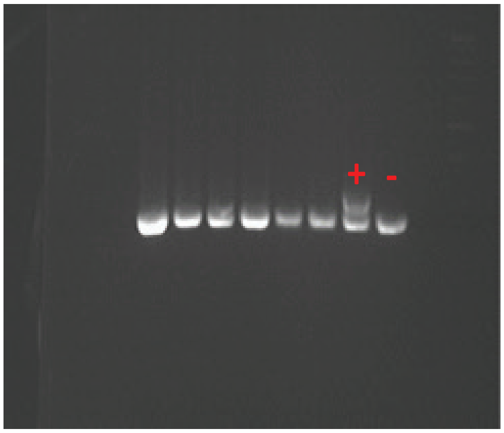

Figure within paper

A

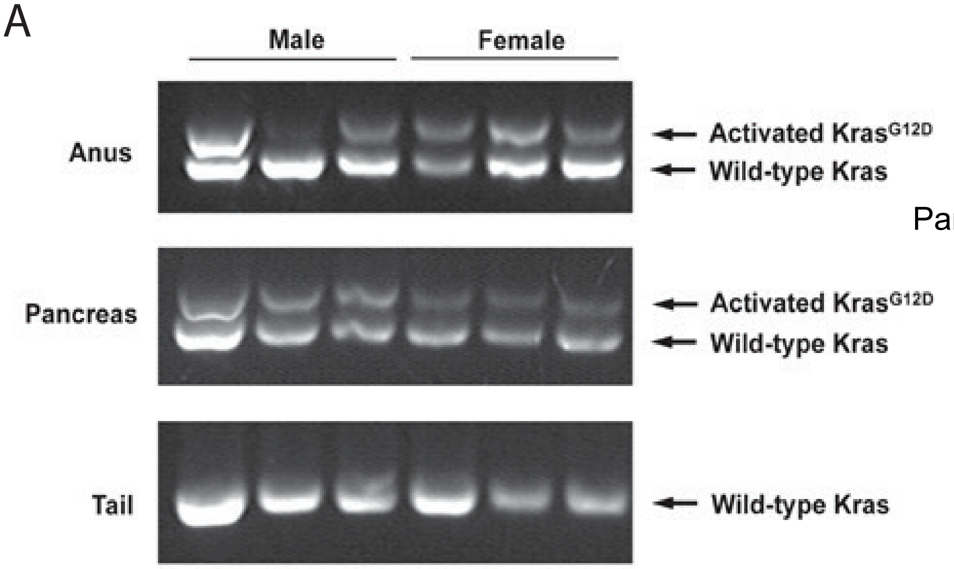

Raw gels for supplemental figure 3
-The supplemental figure is included next to the raw images for clarity as to which part came from which raw gel.

Raw Images

i

Anus

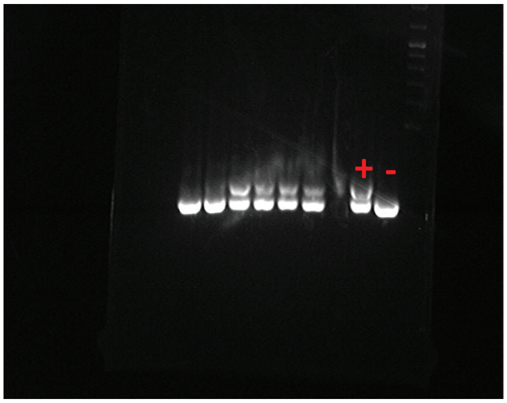

ii

Pancreas

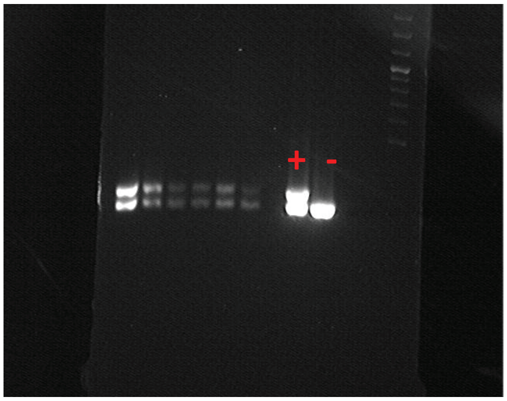

iii

Tail

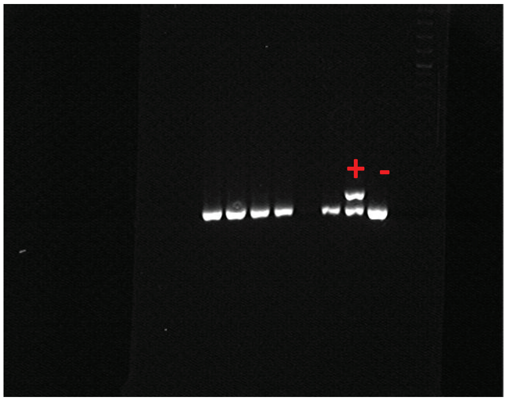

Main Image

A

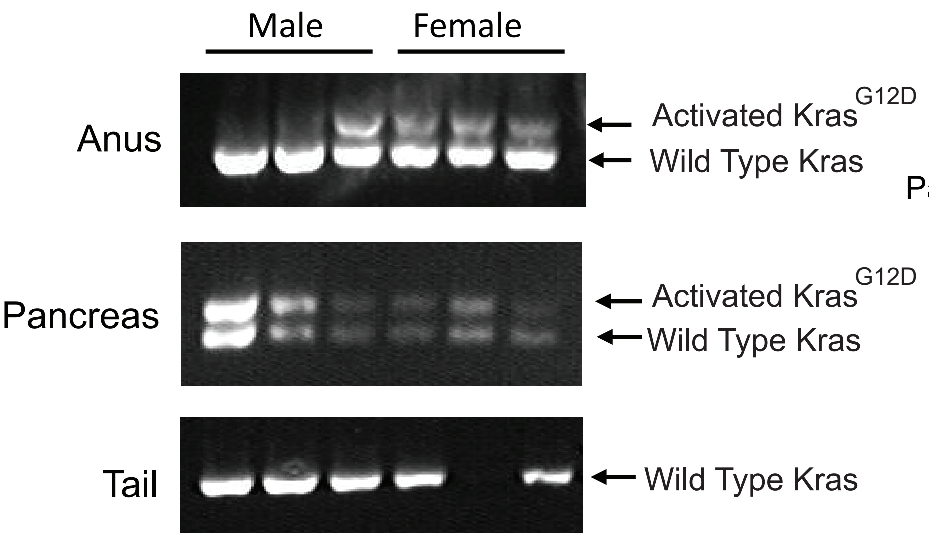

Raw images from supplemental figure 4
-The supplemental figure is included next to the raw images for clarity as to which part came from which raw gel.

Main Image

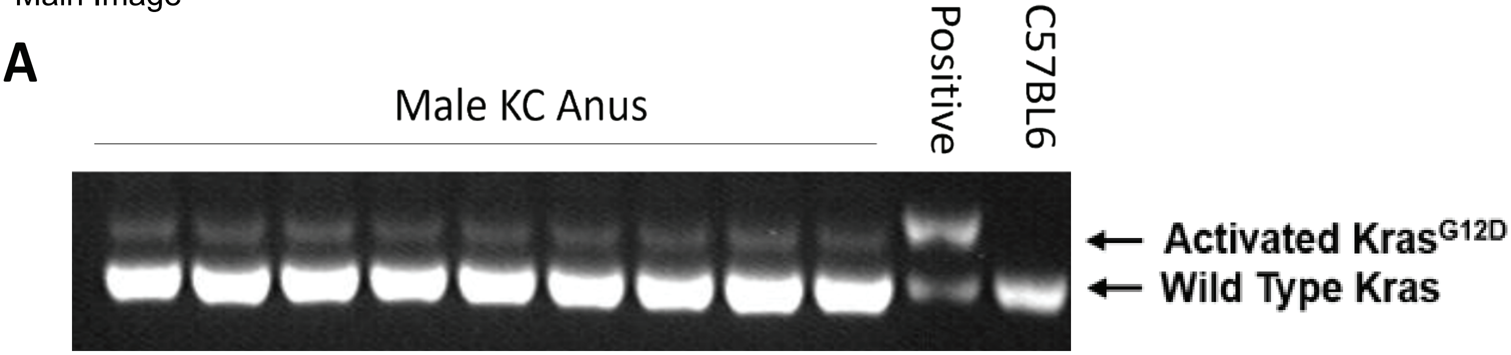

Raw Image

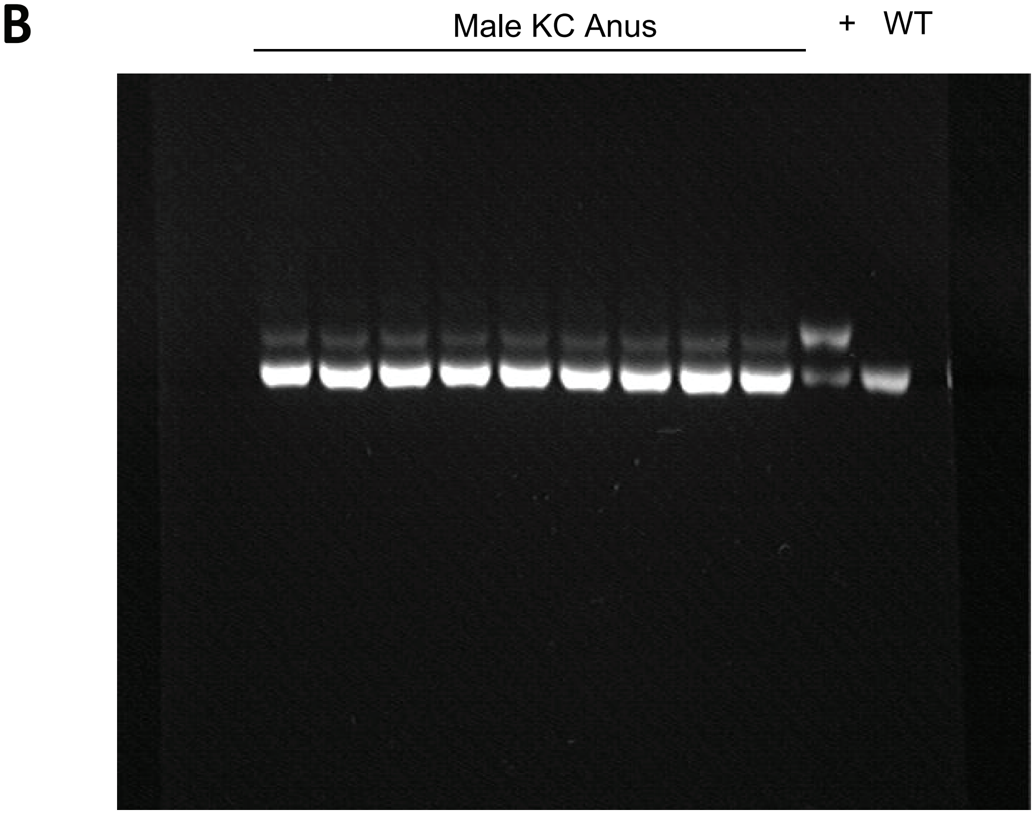

Supplement: S1 Raw images — (PDF) [file pone.0259245.s006.pdf]
